# Accuracy of ROSA Knee System in Bone Cuts Orientation During Total Knee Arthroplasty: An Observational Study

**DOI:** 10.3390/jcm14155205

**Published:** 2025-07-23

**Authors:** Stefano Petrillo, Filippo Migliorini, Giorgio Moretti, Sergio Romagnoli

**Affiliations:** 1 Joint Replacement Department, IRCCS Galeazzi-Sant’Ambrogio Hospital, 20157 Milan, Italy; stefano.g.petrillo@gmail.com (S.P.); giomore95@gmail.com (G.M.); sergio.romagnoli@libero.it (S.R.); 2 Department of Orthopaedic and Trauma Surgery, Academic Hospital of Bolzano (SABES-ASDAA), Via Lorenz Böhler 5, 39100 Bolzano, Italy; 3 Department of Life Sciences, Health, and Health Professions, Link Campus University, Via del Casale di San Pio V, 00165 Rome, Italy

**Keywords:** robotic-assisted arthroplasty, knee, total knee arthroplasty, ROSA, accuracy, persona knee system

## Abstract

**Background**: The ROSA Knee System (Zimmer Biomet, Warsaw, IN, USA) is a robotic system aiming to increase bone resections and component alignment accuracy during TKA. While much is known about its performance in the coronal plane, its accuracy in the sagittal plane remains debated. The present investigation evaluated the system’s accuracy in achieving planned mechanical axis alignment and specific knee angles in both planes. **Methods:** A retrospective analysis was performed on 55 consecutive patients who underwent robotic-assisted TKA using the ROSA Knee System. Data on the medial proximal tibial angle (MPTA), lateral distal femoral angle (LDFA), hip–knee–ankle angle (HKA), tibial slope (TS), and distal femoral flexion (DFF) were collected pre- and post-operatively using the ROSA software. Planned and achieved angles were compared, with deviations greater than 2° and 3° defined as outliers. **Results:** The mean differences between planned and achieved angles for LDFA and MPTA were 0.5° ± 1.00° and 0.3° ± 1.3°, respectively, with less than 10% outliers. The hip–knee angle recorded only a minimal deviation from planned values. In contrast, the TS angle showed a statistically significant difference between planned and achieved values, while no significant difference was found for the DFF angle. The surgeon’s experience did not impact alignment accuracy. **Conclusions:** The ROSA Knee System demonstrates high accuracy in achieving planned alignment in the coronal plane during robotic-assisted TKA, with minimal outliers and reliable predictions for both femoral and tibial angles. However, the ROSA Knee System showed less accuracy in the sagittal plane, particularly for the tibial slope, which did not adversely affect the implant’s stability.

## 1. Introduction

Robotic-assisted total knee arthroplasty (RA-TKA) surgery has gained popularity for improving surgical precision and reducing the percentage of unsatisfied patients [1,2,3]. However, the lack of randomized controlled trials comparing RA with conventional total knee arthroplasty (TKA), or comparative studies including large samples of patients, to date is not reasonable to report the certainty that robotic-assisted surgery is the gold standard in knee arthroplasty. Proper alignment is a major factor in implant longevity and joint function [4]. Radiographs are crucial for preoperative planning and postoperative assessments in TKA, and achieving planned resection angles is essential for considering a robotic system reliable and safe [5,6,7,8]. The ROSA (RObotic Surgical Assistant) Knee System (Zimmer Biomet, Warsaw, IN, USA) is a collaborative robotic and computer device to assist orthopedic surgeons during TKA [9,10,11]. This robotic-assisted device promises to enhance precision by piloting bone resections [12]. Several studies have demonstrated the ability of the ROSA Knee System to achieve specific limb alignment and angles in the coronal plane, such as the medial proximal tibial angle (MPTA), lateral distal femoral angle (LDFA), and hip–knee–ankle angle (HKA) [13,14,15,16]. However, low accuracy and a high percentage of outliers were reported in the sagittal plane, especially concerning distal femur flexion (DFF) and tibial slope (TS) angles [17].

This investigation evaluates the accuracy of the ROSA Knee System in achieving planned MPTA, LDFA, HKA, DFF, and TS by comparing the planned angle values with those obtained from postoperative radiographs three months postoperatively. The authors hypothesized that the percentage of outliers would be less than 10% for each considered measurement.

## 2. Materials and Methods

The present study was performed according to the Strengthening the Reporting of Observational Studies in Epidemiology (STROBE) guidelines [18]. The Ethics Committee of San Raffaele University of Milan approved the present study (ALLCCP, Em. 225-2024). The present study was conducted according to the principles of the Declaration of Helsinki and its later amendments. All patients understood the nature of their treatment and provided written consent to use their clinical and imaging data for research purposes. The inclusion criteria were adults with end-stage knee osteoarthritis stage III or IV according to the Kellgren–Lawrence grading scale, with adverse impact of knee disease on the patient’s quality of life, inadequate response to conservative treatment for at least 6 months, and preoperative flexion contracture of less than 10 degrees. The exclusion criteria were infection, previous ipsilateral knee surgeries, mono-compartmental knee arthritis, ipsilateral hip arthroplasty, revision setting, missing postoperative radiographs, and incomplete ROSA reports.

## 2.1. Surgical Procedures

All surgical procedures were performed by the main author (S.P.) at the Joint Replacement Department, IRCCS Galeazzi-Sant’Ambrogio Hospital, Milan, Italy. All patients received Persona (Zimmer Biomet, Warsaw, IN, USA) ultracongruent cruciate-retaining TKA. All procedures were performed in a highly standardized fashion using a medial midvastus surgical approach. Two femoral pins (3.2 mm diameter) and two tibial pins (3.2 mm diameter) were positioned in the proximal part of the surgical access and in the distal third of the medial tibia. Restricted kinematical alignment and an adjusted mechanical alignment technique were used in the varus and valgus knees, respectively.

## 2.2. Outcomes of Interest

**Figure 1:**
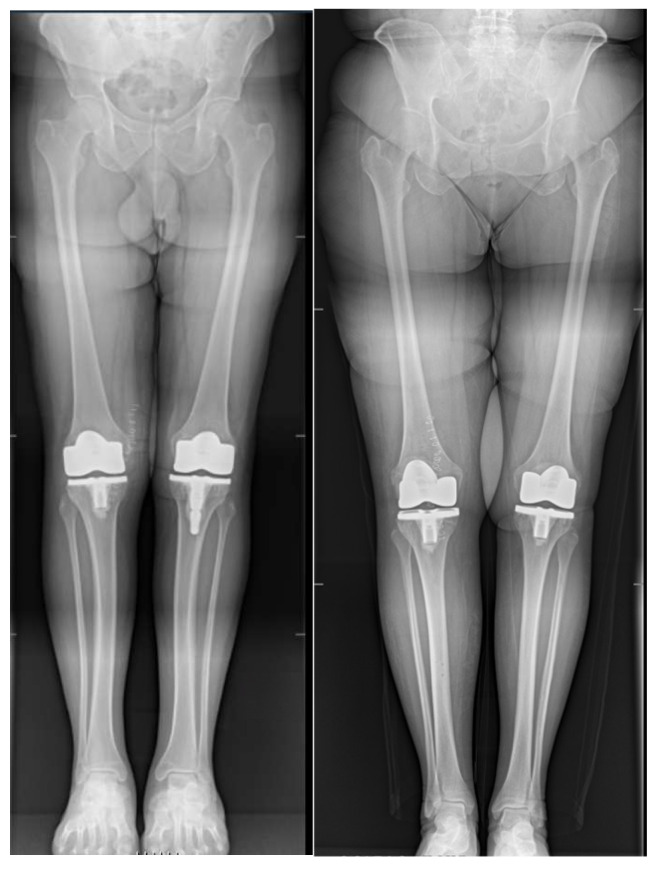
Example of a full-length weight-bearing anteroposterior lower limb radiograph used for measurements.

**Figure 2:**
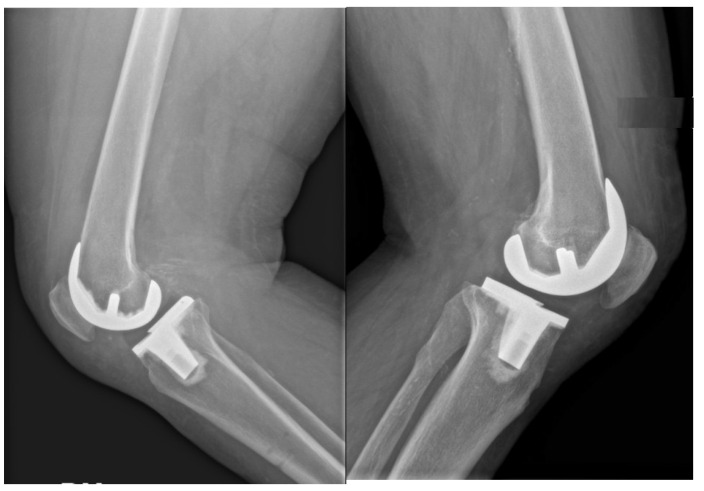
Example of a lateral 90° flexion knee radiograph used for measurements.

**Figure 3:**
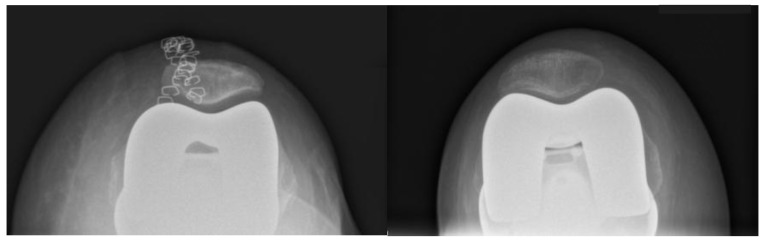
Example of an axial radiograph of the patella (Merchant view) used for measurements.

According to limitations previously highlighted [19], the planned intraoperative MPTA, LDFA, HKA, DFF, and TS angles of bone resections were extracted from the surgical report generated by the ROSA Knee System. Postoperative evaluation of radiographic outcomes was conducted three months postoperatively, and angle measurements were performed, as shown in Table 1. Standardized full-length weight-bearing anteroposterior lower limb radiographs and lateral 90° flexion knee and axial radiographs of the patella were obtained (Figure 1, Figure 2 and Figure 3). Incomplete radiographs because of a lack of weight bearing or incomplete extension warranted the exclusion from the present study. The arithmetic mean was calculated for each measurement performed by the two observers, and the obtained values were used for statistical purposes. Outliers from target angles were considered as follows: MPTA 90° ± 3°, LFDA 90° ± 3°, HKA 180° ± 3°, DFF 3° ± 3°, and TS 7° ± 3.

## 2.3. Data Synthesis

A biomedical statistician performed all the statistical evaluations using the SAS^®^ Version 9.4 software. Descriptive statistics were used to summarize the data and included absolute and relative frequencies for categorical data and means and standard deviations for continuous values. Given the non-normal distribution of the variables considered, the Wilcoxon signed-rank test was used to compare the robotic and radiographic femoral and tibial angles in both sagittal and coronal planes. The accuracy of the ROSA Knee System, expressed by the difference between the robot and the radiograph results, was also estimated. Moreover, the influence of numbers over time on ROSA accuracy was assessed. Values of *p* lower than 0.05 were considered statistically significant.

## 3. Results

### 3.1. Patient Demographics

Sixty-seven consecutive patients with knee osteoarthritis (OA) managed with robotic-assisted TKA between February 2023 and June 2024 were included in the study. In contrast, two (3%) patients were excluded due to incomplete postoperative radiographs or ROSA surgical reports. Considering the first 10 patients as part of the learning curve of the surgeon, the demographic characteristics of the remaining 55 patients enrolled in the study are summarized in Table 2.

### 3.2. Results Synthese

For LDFA and MPTA, the outliers were 2° to 3° in 7.5% and 9.5% of patients, respectively. No outliers higher than 3° were found for LDFA, while the percentage of outliers higher than 3° was 5.7% for MPTA (Table 3).

No significant differences were found between planned (robotic) and achieved (radiographic) results for femoral and tibial coronal angles and HKA (Table 4 and Figure 1).

Moreover, the accuracy of ROSA software prediction was acceptable for both LDFA (r = 0.3) and MPTA (r = 0.4). The mean difference between those planned with robotic software and those measured with radiographs for LDFA and MPTA was 0.53 ± 1.00 and 0.31 ± 1.29, respectively (Table 5).

A statistically significant difference (*p* < 0.0001) was observed for planned and achieved TS angles, while no difference was observed for planned and achieved DFF flexion angles (Table 6; *p* = 0.3576).

Finally, as reported in Table 7, the accuracy in alignment prediction was not influenced by the surgeon’s experience.

## 4. Discussion

According to the main finding of the present investigation, the ROSA Knee System was associated with a percentage of outliers lower than 10% during robotic-assisted TKA. Moreover, outliers greater than 3° were found in only 5.7% of patients for MPTA. The accuracy in the coronal plane is crucial to guarantee the longevity of a TKA [20]. ROSA software can plan femoral and tibial coronal angles with good precision, with a difference between planned and achieved LDFA and MPTA of 0.53 ± 1.00 and 0.31 ± 1.29, respectively. This result agrees with those observed in previously published preclinical and biomechanics studies [17,21,22]. Furthermore, a significant accuracy for the HKA with only 3.6% of outliers was found, confirming results reported by Rossi et al. and Schrednitzki et al. [14,15,23]. Similar results were recently reported by Hax et al., who have demonstrated that most angles in the coronal plane were generally within the target range set for both groups and more frequently for femur than tibia components [24].

Moreover, regarding the evaluation of HKA, the preoperative lower limb axis did not influence the accuracy of the robot in the varus and valgus limbs. Finally, after carefully registering the system and with sufficient experience with the robotic software, acceptable coronal alignment results could be achieved with Persona TKA.

The accuracy of the ROSA Knee System in the sagittal plane is still debated. A good sagittal alignment is crucial to restoring knee flexion and function, significantly influencing patient satisfaction. The ROSA Knee System can be considered inaccurate for the sagittal cuts, reaching percentages of accuracy of 51% within 2° and 77% within 3° for the DFF and 57% within 2° and 74% within 3° for the TS [17]. However, this study identified DFF angles even though there were no differences between planned and achieved angles, while a significant difference was observed in the TS angle. Indeed, the TS angle was lower in radiographs than in the planned robotic software. However, these results usually do not negatively influence the stability of the implant during knee flexion. The saw is not long enough to reach the posterior aspect of the tibia when inserted in the tibial jig, possibility explaining these findings. Another explanation of the result could be that in the case of the large and long tibial plateau, especially in men, the sclerotic bone bends the saw during tibia resection, resulting in reduced TS. Another hypothesis, also supported by Hax et al., is that the reduced TS could result from an excessive anterior positioning of the tibial plateau center landmark during ROSA System registration [24]. The precision and accuracy of a robotic system are significantly related to landmark calibration, which can be prone to individual errors. Furthermore, as also reported by Hax et al., the reasons why the index robotic system demonstrated more accuracy in the coronal plane postoperatively rather than in the sagittal plane remain unclear. In this study, the surgeon chose to position tibial pins in the distal third of the tibia and femoral pins in the proximal portion, a not universally adopted practice. This approach could influence the software’s final alignment predictions and measurements. Finally, according to our results, after excluding the first ten patients as part of the surgeon’s learning curve, the number did not influence the alignment accuracy of the robotic system.

The small sample size and the lack of a formal control group (manual TKA or other robotic systems) impair the reliability of the results of the present investigation [13,14,25,26,27]. Moreover, a cost–benefit analysis comparing robotic-assisted TKA to conventional manual techniques could provide additional insights. The main author performed all surgeries independently in a highly standardized fashion, which might not reflect outcomes in other surgery settings. Although the surgeon had not used ROSA before, he completed four hours of theoretical and three hours of cadaveric lab training during a Zimmer Biomet Institute course before his first robotic-assisted TKA. In our previous study [28], we demonstrated a rapid reduction in surgical time and a high level of accuracy in component size prediction using ROSA, with a learning phase of only ten cases. For this reason, we decided to exclude the first ten patients in the present investigation. Measurement accuracy depends on the quality of radiographic images. Although these were standardized according to institutional protocols, minor errors, particularly in component rotation, cannot be ruled out. Comparing results with one-year postoperative images or computed tomography (CT) scans could address this issue [17]. Strengths of this study are the use of a single implant model, reducing variability in prosthesis positioning, the consistent standardization of perioperative care, including anesthesia and postoperative analgesia, and a stable surgical team, enhancing this study’s reliability.

## 5. Conclusions

The ROSA Knee System demonstrated high accuracy in achieving planned alignment in the coronal plane during robotic-assisted TKA, with minimal outliers and reliable predictions for both femoral and tibial angles. However, the ROSA Knee System showed less accuracy in the sagittal plane, particularly for the tibial slope, which did not adversely affect the implant’s stability. These findings suggest that the ROSA Knee System is reliable in achieving precise alignment in robotic-assisted TKA, though improvements in sagittal plane accuracy may be needed.

## Figures and Tables

**Table 1:** Measurement of coronal and sagittal angles with radiographs. LDFA = lateral distal femoral angle; MPTA = medial proximal tibial angle; TS = tibial slope; DFF = distal femoral flexion.

Radiographic Angle	Radiograph Needed	Measurement Steps	Normal Values
MPTA	Anteroposterior view of the knee (AP)	First line along the tibial plateau, second line tibial mechanical axis	87–90°
LDFA	Anteroposterior view of the knee (AP)	First line along the distal femoral condyles, second line femoral mechanical axis	87–90°
HKA	Full-length weight-bearing AP view of the lower limb, including hip and ankle	First line from the center of the hip to the center of the knee joint, second line from the center of the knee joint to the center of the ankle joint	180° ± 3°
TS	Lateral view of the knee	First line along the tibial plateau, second line perpendicular to the anterior tibial cortex	7° with Persona CR
DFF	Lateral view of the knee	First line along the femoral shaft, second line along the distal femoral condyles	3° with Persona CR

**Table 2:** Demographic characteristics of the patients.

Patient Demographics	Mean ± SD	Value	Range
Age (years)	69.6 ± 8.1	8.1	48–84
Sex	15 male	40 female	n.a.
Body mass index (BMI) kg/m^2^	26.3 ± 4.5	4.5	24–32
Kellgren–Lawrence (KL) grade	grade 3	23	n.a.
	grade 4	30	n.a.
Hip–knee–ankle angle (HKA)	Valgus:		
	−7.1° ± 5.24	17 cases	−1°–−15.5°
	Varus:		
	6.7° ± 4.66	38 cases	0.5°–11.5°

**Table 3:** Alignment outliers.

Parameter	Percentage < 2°	Percentage < 3°
LDFA	92.5%	100%
MPTA	90.5%	94.3%

**Table 4:** Comparison between planned and achieved coronal angle values.

Parameter	Mean ± SD Robot	Mean ± SD Post-Op Rx	*p*-Value
LDFA	90.0 ± 1.12	90.5 ± 0.94	0.09
MPTA	89.0 ± 1.47	89.3 ± 1.03	0.1
HKA	Valgus −1.90° ± 0.66	Valgus −1.86° ± 1.32	0.3
	Varus 1.29° ± 0.92	Varus 1.59° ± 0.81	0.2

**Table 5:** Difference between planned and achieved coronal angle values.

Parameter	Mean ± SD (95% CI)	Min–Max
LDFA	0.53 ± 1.00 (0.26; 0.80)	−1.0; 2.6
MPTA	0.31 ± 1.29 (−0.05; 0.66)	−2.2; 3.5

**Table 6:** Comparison between planned and achieved sagittal angle values.

Parameter	Mean ± SD	Median (IQR)
TS	6.46 ± 1.18	6.5 (1.20)
DFF	3.02 ± 1.07	3.0 (1.30)

**Table 7:** Relationship between case number and alignment accuracy.

LDFA	First Patients	Last Patients	*p*-Value
Patients	10	45	
Mean ± SD	0.31 ± 0.80	0.63 ± 1.07	
Median	0	0.3	0.4141
Min–Max	−0.8–1.8	−1.0–2.6	
95% CI	−0.26; 0.88	0.18; 1.09	
**MPTA**	**First patients**	**Last patients**	***p*-Value**
Patients	10	45	
Mean ± SD	0.42 ± 1.14	−0.08 ± 1.01	
Median	0	0	0.3345
Min–Max	−1.0–2.5	−1.0–2.6	
95% CI	−0.40; 1.24	−2.0; 1.5	

## Data Availability

The original contributions presented in the study are included in the article. Further inquiries can be directed to the corresponding author.

## Abbreviations

The following abbreviations are used in this manuscript:

RA-TKA	Robotic-assisted total knee arthroplasty
TKA	Total knee arthroplasty
MPTA	Medial proximal tibial angle
LDFA	Lateral distal femoral angle
HKA	Hip–knee–ankle angle
DFF	Distal femoral flexion
TS	Tibial slope
OA	Osteoarthritis
CT	Computed tomography
